# Precision Tailoring Quasi-BIC Resonance of a-Si:H Metasurfaces

**DOI:** 10.3390/nano13111810

**Published:** 2023-06-05

**Authors:** Athira Kuppadakkath, Ángela Barreda, Lilit Ghazaryan, Tobias Bucher, Kirill Koshelev, Thomas Pertsch, Adriana Szeghalmi, Duk Choi, Isabelle Staude, Falk Eilenberger

**Affiliations:** 1 Institute of Applied Physics, Abbe Center of Photonics, Friedrich Schiller University Jena, Albert-Einstein-Str. 15, 07745 Jena, Germany; athira.kuppadakkath@uni-jena.de (A.K.); angela.barreda@uni-jena.de (Á.B.); lilit.ghazaryan@uni-jena.de (L.G.); tobias.bucher@uni-jena.de (T.B.); thomas.pertsch@uni-jena.de (T.P.); adriana.szeghalmi@uni-jena.de (A.S.); isabelle.staude@uni-jena.de (I.S.); 2 Institute of Solid State Physics, Friedrich Schiller University Jena, Helmholtzweg 3, 07743 Jena, Germany; 3 Research School of Physics, Australian National University, Canberra, ACT 2601, Australia; kirill.koshelev@anu.edu.au (K.K.); duk.choi@anu.edu.au (D.C.); 4 Fraunhofer-Institute for Applied Optics and Precision Engineering IOF, Albert-Einstein-Str. 7, 07745 Jena, Germany; 5 Max Planck School of Photonics, Hans-Knöll-Straße 1, 07745 Jena, Germany

**Keywords:** quasi-BIC, dielectric metasurface, tailoring, *Q*-factor

## Abstract

The capability of tailoring the resonance wavelength of metasurfaces is important as it can alleviate the manufacturing precision required to produce the exact structure according to the design of the nanoresonators. Tuning of Fano resonances by applying heat has been theoretically predicted in the case of silicon metasurfaces. Here, we experimentally demonstrate the permanent tailoring of quasi-bound states in the continuum (quasi-BIC) resonance wavelength in an a-Si:H metasurface and quantitatively analyze the modification in the *Q*-factor with gradual heating. A gradual increment in temperature leads to a spectral shift in the resonance wavelength. With the support of ellipsometry measurements, the spectral shift resulting from the short-duration (ten minutes) heating is identified to be due to refractive index variations in the material rather than a geometric effect or amorphous/polycrystalline phase transition. In the case of quasi-BIC modes in the near-infrared, resonance wavelength could be adjusted from T = 350 °C to T = 550 °C without affecting the *Q*-factor considerably. Apart from the temperature-induced resonance trimming, large *Q*-factors can be attained at the highest analyzed temperature (T = 700 °C) in the near-infrared quasi-BIC modes. Resonance tailoring is just one of the possible applications of our results. We expect that our study is also insightful in the design of a-Si:H metasurfaces where large *Q*-factors are required at high temperatures.

## 1. Introduction

Optical bound states in the continuum (BICs) are a class of localized states found in the continuum of radiating modes [1]. Since BICs do not couple to the incident radiation, they can be classified according to the cause of the radiation suppression. Symmetry-protected BICs occur when the symmetry of the BIC modes and that of the radiating waves are not compatible [2,3]. Another class of BICs, known as accidental BICs, arises when the continuous tuning of parameters of the system generates the accidental vanishing of the coupling coefficient to the radiative waves [4]. In light of the impossibility of coupling to the incident radiation, BICs are theoretically predicted to have infinite radiative *Q*-factors. In practice, however, such infinite *Q*-factors are unattainable in the samples that can be realized experimentally due to their finite size, absorption or structural disorder, and parasitic scattering. As a result, in realistic scenarios, BICs are converted into leaky modes, referred to as quasi-BICs, which interact with a broad background resonance, resulting in a Fano resonance characterized by a high *Q*-factor and asymmetric lineshape. For symmetry-protected BICs manifested in metasurfaces, quasi-BICs can be observed by breaking their symmetry, thus opening a radiation channel. The higher *Q*-factor of these states compared to Mie modes makes quasi-BICs interesting in many applications such as refractive index sensing [5,6], imaging [7,8], electromagnetically induced transparency generation [9], energy conversion [10], non-linear optics [11] (e.g., enhancing the second-harmonic conversion efficiency [3,12]), or enhancing the emission from quantum emitters [13,14], among others [15,16]. The *Q*-factor of the asymmetric quasi-BIC structures is known to be dependent on the asymmetry parameter [17]. Given the existence of high *Q*-factor and hence narrow-band resonances, whose wavelengths strongly depend on minute geometrical details, a targeted fabrication of a specific resonance wavelength is extremely challenging, even for state-of-the-art lithography tools. The size, as well as the arrangement of the meta-atoms in the metasurface, can modify the functioning of the nanostructure. Different phases emerging from the perturbation in the distribution of the individual units are discussed by Rahimzadegan et al. [18]. A decline in the radiative *Q*-factor and spectral shift is illustrated when the variations in the geometric parameter of a BIC unit cell are simulated in order to address the limitations due to fabrication tolerance [19]. The findings from the above-mentioned studies signify the requirement for a technique to mitigate fabrication errors. We, therefore, investigate post-fabrication transient heating as a strategy for permanently tailoring quasi-BIC resonances towards a specific target wavelength.

The high thermo-optic coefficient of crystalline silicon (c-Si) was previously explored for reversible tuning of magnetic dipole and electric quadrupole modes of silicon nanodisks in the infrared wavelengths for temperatures ranging from T = 20 °C to T = 300 °C [20]. Further, image contrast manipulation was achieved on a Yin-Yang pattern made of two types of c-Si nanoresonators by utilizing similar effects [21]. In a related study, c-Si and germanium meta-atoms were found to have spectral shifts in their electric and magnetic dipole modes in the far-infrared wavelengths when heated in the range from T = −193.15 °C to T = 526.85 °C [22]. The temperature dependence of electric and magnetic Mie modes [23], Fano resonances [24], and quasi-dark resonances [25] of infrared-active c-Si metasurfaces was presented as a tool for temperature sensing. Red-shift in the Mie modes of a c-Si nanosphere in the visible wavelength was reported when treated with an electron beam [26]. Laser-assisted post-processing based on ablation/heating was tested on low *Q*-factor-dipolar resonances in amorphous silicon by creating structural and material modifications [27]. In this work, we experimentally demonstrate temperature as a tool for trimming the quasi-BIC resonance excited in differently designed hydrogenated amorphous silicon (a-Si:H) metasurfaces by employing the temperature-induced refractive index modification of a-Si:H. We illustrate the trimming functionality in different spectral regions: visible and near-infrared (NIR).

Hydrogenated amorphous silicon is preferred over c-Si for realizing high *Q*-factor quasi-BIC resonances in the visible and NIR wavelengths due to its lower losses in the wavelengths ranging from 650 nm to 1100 nm. The temperature-dependent optical properties of a-Si:H is different compared to that of c-Si [28]. The heating process can alter the distribution of hydrogen bonds, crystallinity, and structural properties leading to variations in its refractive index. Prolonged heating of a-Si:H metasurface with the direct increment of temperature from T = 22 °C to T = 700 °C was found to result in amorphous to polycrystalline phase transition and a sharp decrement in the *Q*-factor [29]. The current study is different because here, we consider intermediate heating steps between T = 22 °C and T = 700 °C as well and for a shorter duration of heating (ten minutes). We investigate the modifications in the quasi-BIC modes after each heating stage and demonstrate that the heat-induced modifications can be utilized for tailoring the quasi-BIC resonance in the metasurface. The observed refinement in the functionality of the metasurface is permanent, i.e., the heat-induced effects are not reversed when cooled to room temperature. Considering the irreversibility of the changes, and due to the long-term stability generally observed in the a-Si:H film and nanostructures, we assume that the thermal modifications would persist for a long duration. However, this can be experimentally investigated in a future work. The thermal tunability (from T = −3.15 °C to T = 116.85 °C) of a strontium titanate layer was utilized for tuning the resonance in a LiTaO${}_{3}$ metasurface showing quasi-BIC resonance at 481.75 μm wavelength [30]. However, this is conceptually similar to the technique of altering the refractive index of the surrounding medium for tuning the resonance as in the case of liquid crystal-assisted tuning methods [31,32,33], or fine-tuning the resonant conditions by the deposition of a few Angstrom thin films [34]. The direct heating of optical quasi-BIC metasurfaces composed of a-Si:H has not been explored in a larger temperature range yet. The main advantage of temperature tuning over phase change materials is that the intermediate refractive index between different phases is accessible, whereas it is difficult to achieve that functionality for phase-change materials [35,36].

## 2. Materials and Methods

The plasma-enhanced chemical vapor deposition (PECVD) technique was used to grow hydrogenated amorphous silicon film on a fused silica substrate [37]. A positive electron beam resist (ZEP520A) was spin-coated on top of the a-Si:H layer. Electron beam lithography (Raith150) was availed to write the pattern. Then, the sample was processed in ZED-N50, followed by the aluminium deposition and lift off process. The patterned aluminium layer functions as a hard mask for dry etching. The pattern was transferred on the a-Si:H film through inductively coupled plasma reactive ion etching (Oxford Plasmalab System100, Oxford Instruments, Yatton, UK). A mixture of phosphoric acid, nitric acid, and acetic acid was used to remove the remaining aluminium from the nanostructure.

Spectroscopic ellipsometry (M2000, J. A.Woollam, Lincoln, NE, USA) was utilized to measure the thickness and refractive index of the samples. The angle of incidence (AOI) available in the instrument is 75°. The Cody–Lorentz model was used to fit the data in the range of 400–900 nm in the CompleteEASE software (version 4.48).

Transmission spectra were measured using a microscope setup customized for linear transmission spectra measurements. The microscope (Carl Zeiss Microscopy GmbH, Jena, Germany) was equipped with a halogen light source. Nearly plane wave incidence was ensured using Koehler illumination. An Andor spectrometer (Oxford Instruments, Yatton, UK) equipped with a cooled CCD detector was used for the spectra measurements.

The Raman spectra measurement was performed under 530 nm laser excitation. The instrument is a confocal Raman microscope commercially available from Renishaw (inVia^TM^ Raman Microscope, Renishaw, Wotton-under-Edge, UK). The excitation and collection were carried out using a 20× microscope objective.

The finite-difference time-domain (FDTD) method was implemented in the commercial software Lumerical (version 2018a) [38] to perform the simulations. For simulating the transmission spectra, a unit cell of the metasurface was considered with periodic boundary conditions along the *x*- and *y*-axis and perfectly matched layers in the *z*-axis. The structure was illuminated by a plane wave source. A monitor in the silica substrate was used to obtain the transmission spectrum.

## 3. Results and Discussion

**Figure 1:**
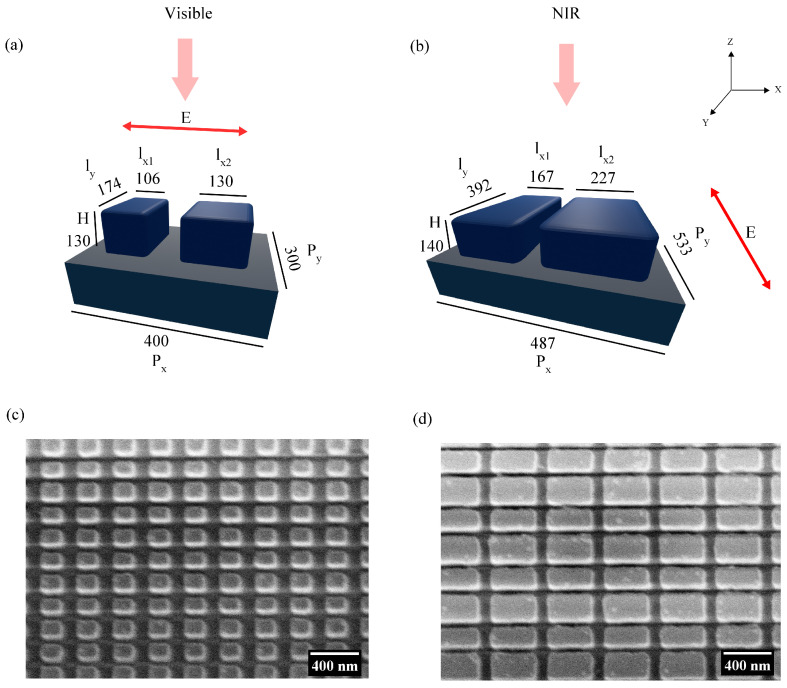
An illustrative of the (**a**) visible metasurface and the (**b**) NIR metasurface with all lengths represented in units of nanometers. The double-sided arrow indicates the polarization, and the single-sided arrow on top of the schematic indicates the propagation direction of the incoming beam. Scanning electron microscope (SEM) image of the (**c**) visible metasurface and the (**d**) NIR metasurface. Note that the orientation of the SEM images in (**c**,**d**) is at 90° angle with respect to the schematics in (**a**,**b**).

Two different a-Si:H metasurface designs are used for studying the effect of gradual heating on quasi-BIC resonances in visible and NIR spectral regions. Henceforth, these designs will be denoted as ’visible’ and ’NIR’ structures. At room temperature, the resonance wavelength of the visible metasurface is λ= 683 nm and that of the NIR metasurface is λ= 825 nm. They consist of asymmetric a-Si:H bars with asymmetry parameters of α= 0.18 and α= 0.26 for the visible and the NIR structure, respectively (asymmetry parameter α is defined as the ratio of the difference in the lengths of the asymmetric bars (δW) to the length of one of the bars (W) [17]). The quasi-BIC resonance of the visible metasurface structure can be excited with horizontally polarized radiation and that of the NIR metasurface structure with vertically polarized radiation. The incident wave propagates along the negative direction of the *z*-axis as represented in the schematics in Figure 1. The parameters shown in the schematics (Figure 1a,b) are the values for which the simulated transmission spectra agree well with the experimental transmission spectra at room temperature. The scanning electron microscope (SEM) images of the visible and the NIR metasurfaces are provided in Figure 1c,d, respectively.

**Figure 2:**
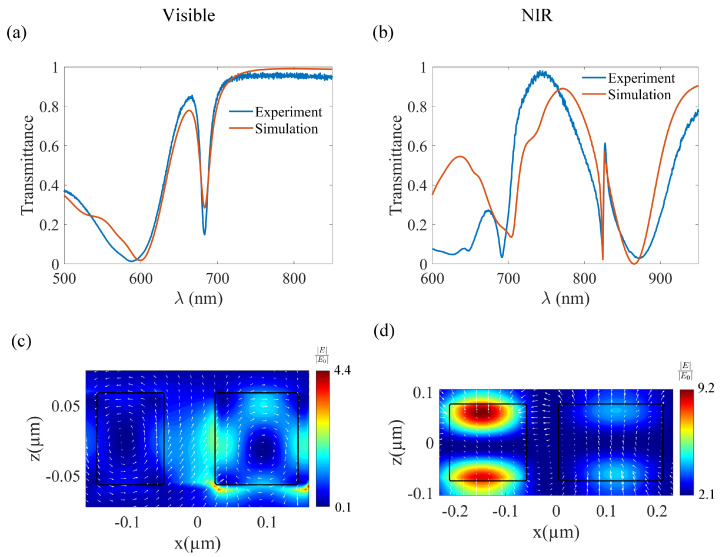
Transmission spectra of the (**a**) visible metasurface and the (**b**) NIR metasurface. The blue and red legends correspond to the measured and simulated spectra, respectively. The normalized electric field intensity map (at the XZ plane through the center of the bars) of the (**c**) visible metasurface (λ = 683 nm) and the (**d**) NIR metasurface (λ = 823 nm). The arrows represent the polarization vector of the Electric fields. The outline of the a-Si:H bars is represented as black rectangles.

The pre-heating transmission spectra of the metasurface structures were measured at room temperature (see Figure 2a,b). A customized linear transmission microscope setup was used for the transmission measurements. The measured transmission spectra were in agreement with the simulations. The experimental *Q*-factors at room temperature were calculated to be *Q =* 54 (visible quasi-BIC) and *Q =* 287 (NIR quasi-BIC). The *Q*-factors values calculated from the simulation results are *Q =* 36 (visible) and *Q =* 295 (NIR), respectively. The *Q*-factors were obtained by fitting the respective data set using Fano fitting. The variations in the simulated and experimental *Q*-factor might be due to fabrication imperfections or slight variations in the parameters at different regions of the metasurface. The above mentioned values are the total *Q*-factors. The total *Q*-factor ($Q_{tot}$) is related to the radiative ($Q_{R}$) and the non-radiative *Q*-factors ($Q_{NR}$) by the equation ${Q_{tot}}^{-1}={Q_{R}}^{-1}+{Q_{NR}}^{-1}$. The radiative *Q*-factor can be calculated from the simulations by assuming zero dissipative losses. At room temperature, the radiative *Q*-factor value of the visible metasurface is 82.4 and that of the NIR metasurface is 459. The non-radiative *Q*-factors estimated based on the $Q_{tot}$ and the $Q_{R}$ values are $Q_{NR}$(visible)=63.9 and $Q_{NR}$(NIR)=825.6. This indicates that the radiative and the non-radiative decays are comparable in the case of the visible metasurface, whereas the radiative decay is weaker in the case of the NIR metasurface. The radiative *Q*-factors computed for higher temperatures are enlisted in the Supplementary Section S5 (Tables S1 and S2).

The near-field map of the electric field enhancement is plotted in Figure 2c,d. The enhancement factor is determined by scaling the near-field map of the meta-atom with respect to that of the unstructured substrate under plane wave illumination.

### Permanent Tailoring of Resonance Wavelength

**Figure 3:**
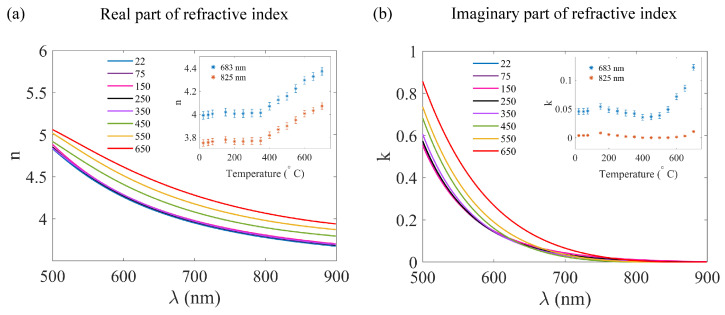
(**a**) Real part of the refractive index (n) plotted as a function of the wavelength for different temperatures (see legend). (**b**) Imaginary part of the refractive index (k) as a function of the wavelength for different temperatures. The insets show the n/k values versus the temperature at the quasi-BIC resonance wavelengths. The error bar depicts the standard error in the measurement. Note that the data for some temperatures are omitted for better visualization (complete data is available in the Supplementary Figure S1).

The metasurface structures were subjected to a temperature cycle, where they were gradually heated for ten minutes (ramp rate = 30 °C/min) from room temperature (T = 22 °C) to T = 700 °C in 50 °C steps. Then, they were allowed to cool to room temperature after each heating cycle. The refractive index was obtained by conducting ellipsometry measurements on an a-Si:H layer of 530 nm thickness deposited on a fused silica substrate. This unstructured film was heated simultaneously with the metasurface structures. The evolution of the real and imaginary parts of the refractive index (n and k) with the temperature increment is presented in Figure 3. Some data sets are avoided in the plots in Figure 3a,b for the convenience of visualization, the complete data can be found in the Supplementary Figure S1. The insets in Figure 3a,b show the n and k values at the resonance wavelengths (λ = 683 nm and λ = 825 nm) with respect to the temperature. From Figure 3a, the real part of the refractive index (n) is observed to grow linearly with the temperature for temperatures above T = 350 °C. The increment in the n-value was about 0.4 in the wavelength range of λ = [650–900] nm. Therefore, the resulting differences in the optical performance are the same for both metasurface structures. Figure 3b shows a dissimilar behavior of the k-value with the rise in temperature, depending on the considered wavelength. In particular, for the visible resonance, the rise of k values with the temperature is evident when annealed above T = 350 °C. However, for the NIR resonance, the k-value increment with the temperature is only manifested for temperatures higher than T = 600 °C. The absorptive losses are higher in the visible range because this is closer to the band-edge of a-Si:H. To better establish comparisons, we have obtained the k-values of the refractive index for both wavelengths of interest at T = 350 °C and T = 700 °C. For λ = 683 nm, the parameters are k = 0.04 at 350 °C and k = 0.12 at 700 °C. For λ = 825 nm, the parameters are k = 0.001 at 350 °C and k = 0.01 at 700 °C.

**Figure 4:**
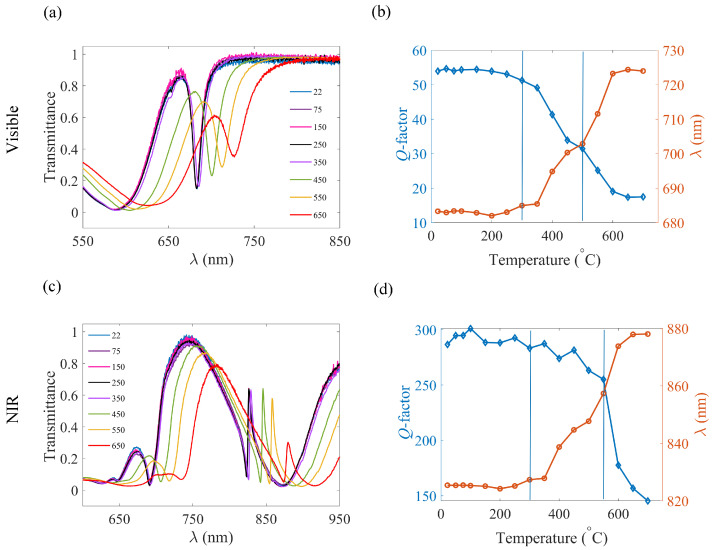
(**a**) Transmission spectra of the visible quasi-BIC structure after heating at different temperatures. (**b**) *Q*-factor and resonance wavelength of the visible quasi-BIC structure as a function of the temperature. (**c**) Transmission spectra of the NIR quasi-BIC structure after heating at different temperatures. (**d**) *Q*-factor and resonance wavelength of NIR quasi-BIC structure as a function of the temperature. The two vertical lines in (**b**,**d**) mark the temperature range over which tailoring can be performed with an acceptable value of the *Q*-factor. The data corresponding to some temperatures are omitted in Figures (**a**,**c**) for better visualization (complete data is given in the Supplementary Figure S2).

The transmission spectra of the visible and NIR quasi-BIC structures after subsequent heating steps are represented in Figure 4 along with a plot of the resonance wavelength and the *Q*-factor against the temperature (the measurements corresponding to some temperatures are omitted in Figure 4a,c for better visualization, the complete data can be found in the Supplementary Figure S2). The resonance wavelength was not affected until T = 350 °C. Above T = 350 °C, a linear shift was observed in the resonance. This observation agrees reasonably well with the simulated transmission spectra provided in Supplementary Figure S3. The slight dissimilarities originate from the measurement inaccuracy in the ellipsometry data. This red-shift can be explained as a result of the increase in the real part of the refractive index (n) plotted in Figure 3a. The resonance wavelength of the visible quasi-BIC structure can be modified from 683 nm to 725 nm by increasing the temperature from T = 400 °C to T = 600 °C, as shown in Figure 4a,b. Similarly, the resonance wavelength of the NIR quasi-BIC structure can be trimmed from 825 nm to 875 nm (Figure 4c,d). A comparison of the experimental and simulated transmission spectra at the lowest and highest temperatures considered are shown in Supplementary Figure S4. For both metasurface designs, the spectral shift is around Δλ = 50 nm. This behavior is expected due to the same modification of the real part of the refractive index with the temperature at the two wavelengths of interest as observed from the ellipsometry measurements (inset of Figure 3a). The amplitude of the resonances declines and they become wider at higher temperatures. We attribute this effect to the increase in the losses (imaginary part of refractive index) with heating. This observation is in accordance with a former study on Mie resonance in c-Si nanospheres, where the rise in the imaginary part of the refractive index caused the broadening of the electric quadrupole and magnetic hexapole modes [26]. In the case of a-Si:H, the higher k-values can be due to reduced hydrogen content and a larger phonon population at elevated temperatures. The absorptive losses are hard to quantify with very high accuracy from the thin a-Si:H film. However, since each measurement is prone to the same measurement error, the overall trend is still reliable within the error limit.

The broadening of the resonances causes a reduction in the *Q*-factor. This decrement is observed for temperatures higher than T = 350 °C for the visible (see Figure 4b) and T = 550 °C for the NIR (see Figure 4d) quasi-BIC structures. Specifically, the *Q*-factor declines from 54 to 17 for the visible structure and 287 to 145 for the NIR quasi-BIC structure when the temperature is increased from room temperature to T = 700 °C. The decrease in the *Q*-factor is also reflected in the field enhancement factors (Supplementary Figure S6) because of the reduction in the electromagnetic energy inside the resonator. It is demonstrated that the reduction in the *Q*-factor is less prominent in the case of the NIR quasi-BIC structure (49% reduction with respect to its initial value versus 68% for the visible quasi-BIC structure) because the increase in the imaginary part of the refractive index with the temperature is much less at the NIR spectral region. The k-value increment is 0.08 around 683 nm, whereas the increment is around 0.009 at 825 nm. A low *Q*-factor at high temperatures is a limitation in using the full potential of the temperature-based modification of the resonance wavelength for the visible quasi-BIC structure. However, the NIR quasi-BIC structure still has a *Q*-factor of 145 even after exposing it to T = 700 °C. The resonance wavelength can be tailored while maintaining an appreciable *Q*-factor in the range of temperatures marked by the lines in Figure 4b,d. From the experimental results, we can conclude that for temperatures higher than 600 °C, the changes in the resonance wavelength and the *Q*-factor can be considered negligible. Plots of the *Q*-factor and the resonance wavelength calculated from the simulated transmission spectra are illustrated in the Supplementary Figure S5.

**Figure 5:**
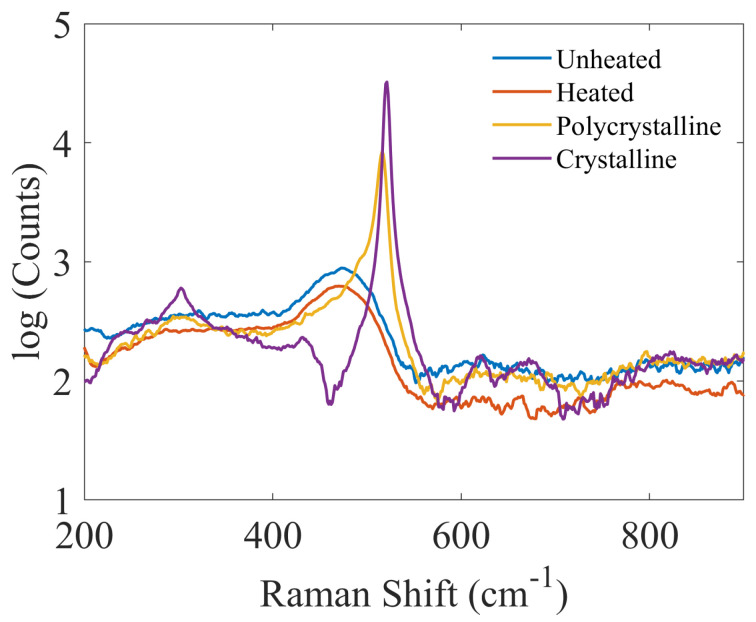
Raman spectra of the unheated, heated, polycrystalline, and crystalline silicon. (Note that the *y*-axis values are in the logarithmic scale for better visualization of the measurements from a-Si:H).

A phase transition from amorphous to polycrystalline can happen above 700 °C when annealed for hours. However, that is not the case in our experiment, where brief-duration heating is implemented. This can be observed from the Raman spectra measurements shown in Figure 5. The Raman spectra of the unheated and heated samples are identical, except for a reduction in the counts of the heated sample. A shift of the peaks towards the signature peak of c-Si was reported in the case of laser-assisted post-processing [27]. In view of these results, we attribute the red-shift of the resonance wavelength in our experiment to a temperature-assisted refractive index modification rather than a phase transition.

The temperature-dependent resonance wavelength of high *Q*-factor quasi-BIC modes can be used for tailoring the metasurface to a desired spectral window permanently.

## 4. Conclusions

Gradual and short-duration heating can be considered as an experimental-feasible tool to tailor the resonance wavelength of high *Q*-factor quasi-BIC modes excited in a-Si:H metasurfaces. In this study, we compared the transmission spectra of metasurfaces exhibiting quasi-BIC resonances in visible and NIR spectral regions after heating from room temperature (T = 22 °C) up to T = 700 °C. The resonance wavelengths of the metasurfaces show a red-shift of 42 nm (visible quasi-BIC structure) and 50 nm (NIR quasi-BIC structure) in the temperature range from T = 350 °C to T = 600 °C. The ellipsometry measurements evidence a gradual progression in the real (n) and imaginary (k) parts of the refractive index values of a-Si:H with the rise in temperature. This variation in the optical properties causes the red-shift of resonance and around a 50% reduction in the *Q*-factor. The lowering of the *Q*-factor is detrimental to the device’s performance. For that reason, it is required to design metasurfaces with high *Q*-factors at room temperature. In this regard, the NIR quasi-BIC structure has a higher *Q*-factor compared to the visible quasi-BIC structure at room temperature. In addition, the increase in the imaginary part of the refractive index, responsible for the decrease in the *Q*-factor with the rise in temperature, is smaller for the NIR than for the visible spectral region. Therefore, it is possible to achieve a large *Q*-factor value of 145 for the NIR quasi-BIC structure even after the heat treatment. Alternatively, localized heating using a laser or electron beam can also be considered as another way of trimming the quasi-BIC resonances of all-dielectric metasurfaces. The thermally-induced modifications in the optical properties can be further exploited in other devices composed of a-Si:H, as well as in metasurfaces fabricated from different materials.

## Data Availability

The data presented in this study are available on request from the corresponding author.

## Supplementary Materials

The following supporting information can be downloaded at: https://www.mdpi.com/article/10.3390/nano13111810/s1, Figure S1: (a) The real and (b) the imaginary parts of the refractive index determined from ellipsometry measurements. The legends denote the heating temperature. Figure S2: Transmission measurements of the (a) visible metasurface and the (a) NIR metasurface at different temperatures. Figure S3: The simulated transmission spectra of the (a) visible metasurface and the (b) NIR metasurface. Figure S4: The simulated and experimental transmission spectra at the lowest and highest analyzed temperatures (T = 22 °C and T = 700 °C) in the case of the (a) visible metasurface and the (b) NIR metasurface. The solid curves correspond to the temperature T = 22 °C and the dotted curves correspond to the temperature T = 700 °C. The blue and orange legends represent the experimental and simulated results. The arrow indicates the red-shift of the resonance wavelength at the high temperature. Figure S5: Resonance wavelength and *Q*-factor versus temperature in the case of the (a) visible metasurface and the (b) NIR metasurface. The ‘∘’ represents the experimental result and the ‘*’ represents the simulated result. The error limits are indicated as the vertical lines on the data points. Figure S6: The near-field Electric field map (in the XZ plane through the center of the bars) at T = 22 °C of the (a) visible metasurface and the (b) NIR metasurface. Near-field Electric field map at T = 700 °C of the (c) visible metasurface and the (d) NIR metasurface. Table S1: Radiative and non-radiative contributions to the *Q*-factor of the visible metasurface at different temperatures. Table S2: Radiative and non-radiative contributions to the *Q*-factor of the NIR metasurface at different temperatures. References [11,16,38] are cited in the Supplementary Materials.
