# Supplementary material for: Precision Tailoring Quasi-BIC Resonance of a-Si:H Metasurfaces
Source: Nanomaterials (Basel). 2023 Jun 5;13(11):1810. doi: 10.3390/nano13111810 (PMC10255339; doi:10.3390/nano13111810)
Supplement: Supplementary file 1 [file nanomaterials-13-01810-s001.zip › nanomaterials-2398185-supplementary.pdf]

Supplementary Materials: Precision Tailoring quasi-BIC resonance of a-Si:H metasurfaces

Athira Kuppadakkath [1], Ángela Barreda [2,1], Lilit Ghazaryan [1], Tobias Bucher [2,1], Kirill Koshelev [3], Thomas Pertsch [1,4,5], Adriana Szeghalmi [1,4,5], Duk Choi [3], Isabelle Staude [2,1] and Falk Eilenberger [1,4,5,*]

S1. Ellipsometry measurements

The real and imaginary parts of the refractive index of an unstructured a-Si:H film were determined using ellipsometry measurement at room temperature after subsequent heating steps. The supplementary Figure S1 is complementary to Figure 3 in the manuscript, where the data for alternate heating steps were not plotted for better visualization. Figure S1a shows the real part of the refractive index (n) as a function of wavelength and Figure S1b shows the imaginary part of the refractive index (k) as a function of the wavelength corresponding to different temperatures.

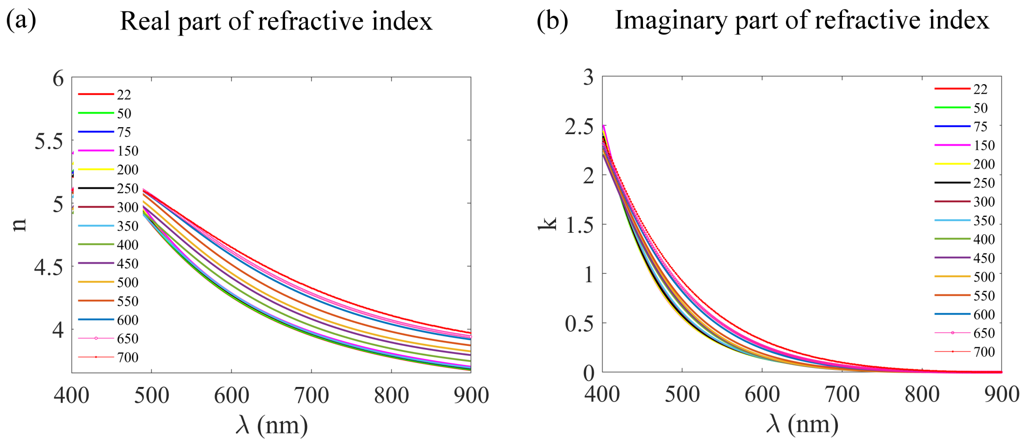

Figure S1. (**a**) The real and (**b**) the imaginary parts of the refractive index determined from ellipsometry measurements. The legends denote the heating temperature.

S2. Measured transmission spectra

After annealing the metasurface for ten minutes at different temperatures, the sample was allowed to cool to room temperature and then the transmission spectra were measured. The supplementary Figure S2 is complementary to Figure 4 in the manuscript. The complete data set of transmission measurements for the visible metasurface is plotted in Figure S2a and that for the near-infrared (NIR) metasurface is plotted in Figure S2b. As mentioned in the manuscript, the two types of metasurfaces are denoted as 'visible' and 'NIR' based on their resonance wavelength at room temperature. The transmission spectra measurements of the visible and NIR metasurfaces at room temperatures exhibit the quasi-bound states in the continuum (BIC) resonance at λ = 683 nm, and λ = 825 nm respectively.

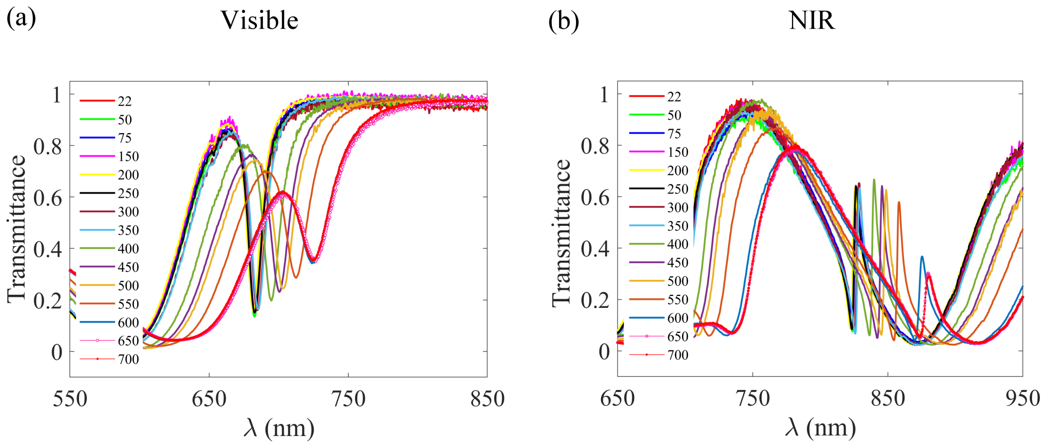

Figure S2. Transmission measurements of the (**a**) visible metasurface and the (**b**) NIR metasurface at different temperatures.

S3. Simulated transmission spectra

The transmission spectra of the visible and the NIR metasurfaces were simulated [1] for different temperatures corresponding to the measurements (Figure S3b). The optical constants (n, and k) obtained from the ellipsometry measurements were used for the simulations. The simulated transmission spectra are comparable to the measured transmission spectra. There can be variations in the simulated transmission spectra from the measured spectra depending on the accuracy of the ellipsometry results. The standard error in the ellipsometry measurements is around 0.03 (for both types of metasurfaces) in the case of the real part of the refractive index, and 0.005 for the imaginary part of the refractive index in the visible region (0.0008 in the NIR region). This error causes the deviation between the measured and simulated spectra. Unlike the experimental observation, the simulated transmission spectra are different for T = 650 °C and T = 700 °C. However, from the experimental results for the metasurface, we can conclude that for temperatures higher than 600°C, the changes in the resonance wavelength and the *Q*-factor can be considered negligible. This indicates that the structure metasurface has a slightly different response to the temperature changes compared to the planar a-Si:H film at very high temperatures.

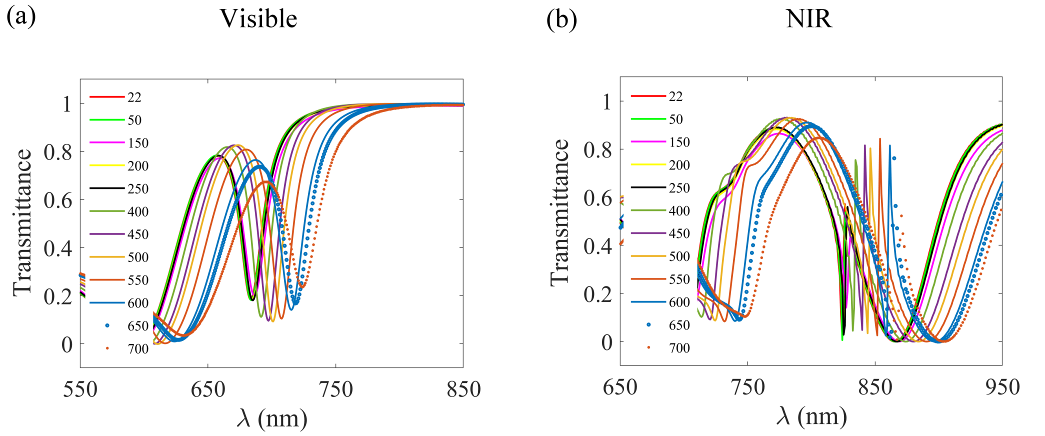

Figure S3. The simulated transmission spectra of the (**a**) visible metasurface and the (**b**) NIR metasurface.

S4. Comparison of the experimental and simulated results at T = 22 °C and T = 700 °C

A comparison of the simulated and experimental transmission spectra at the lowest (T = 22 °C) and the highest (T = 700 °C) analyzed temperatures are provided in supplementary Figure S4. The experimental and simulated transmission spectra agree reasonably well at low and high temperatures for both the visible (Figure S4a) and the NIR (Figure S4b) metasurfaces.

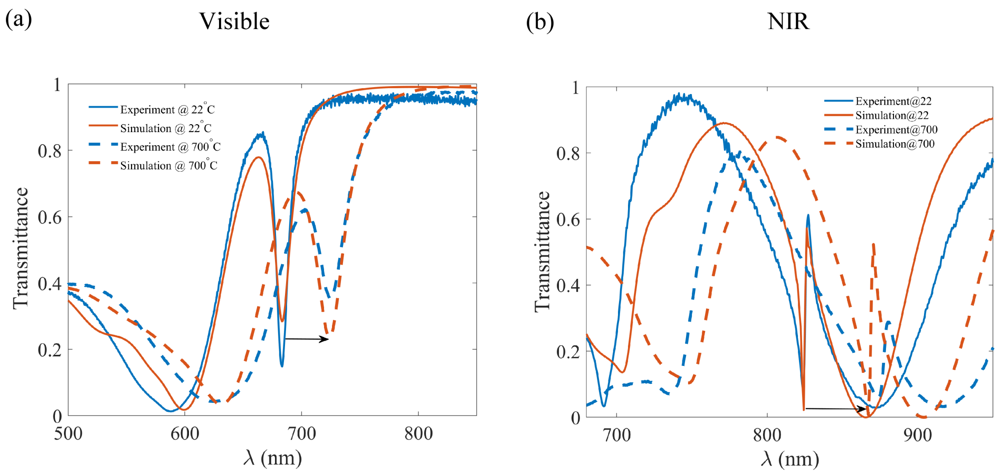

Figure S4. The simulated and experimental transmission spectra at the lowest and highest analyzed temperatures (T = 22 °C and T = 700 °C) in the case of the (**a**) visible metasurface and the (**b**) NIR metasurface. The solid curves correspond to the temperature T = 22 °C and the dotted curves correspond to the temperature T = 700 °C. The blue and orange legends represent the experimental and simulated results. The arrow indicates the red-shift of the resonance wavelength at the high temperature.

S5. Quasi-BIC resonance from the simulations

The simulated transmission spectra at different temperatures were fitted with the Fano formula and the resonance wavelengths were extracted. Supplementary Figure S5 shows the resonance wavelength and the Q-factor as a function of temperature as per the simulation results. The simulated results are represented by a circle ('$\circ$'), along with the standard error, and the values obtained from the experimental observation are marked by a star ('$*$') for the purpose of comparison. As observed in the experiment, the resonance wavelengths shift to larger wavelengths for both the visible (Figure S5a) and the NIR (Figure S5b) metasurfaces. According to this analysis, the wavelength tailoring for the visible quasi-BIC resonance is from 683 nm to 723 nm, which agrees well with the experimental observation (683 nm to 723 nm). In the case of NIR metasurface, the resonance wavelength can be varied from 825 nm to 867 nm (whereas in the experimental observations, this range is from 825 nm to 875 nm). There is remarkable agreement in the wavelengths obtained from the experimental observations and simulations. The calculated Q-factors however show slight differences compared to the experiment. These variations result from the fact that the simulations correspond to an ideal case, where the parameters of the nano bars are the same throughout the sample. But there can be local variations in these parameters at different parts of the metasurface due to fabrication challenges. Besides, the error in the ellipsometry is also reflected in the values of the Q-factor. For example, the slight curvature in the k-values seen in the inset of Figure 3(b) of the main manuscript is reflected as a curvature in the Q-factor values (calculated from simulation) in the temperature range 400–600 °C.

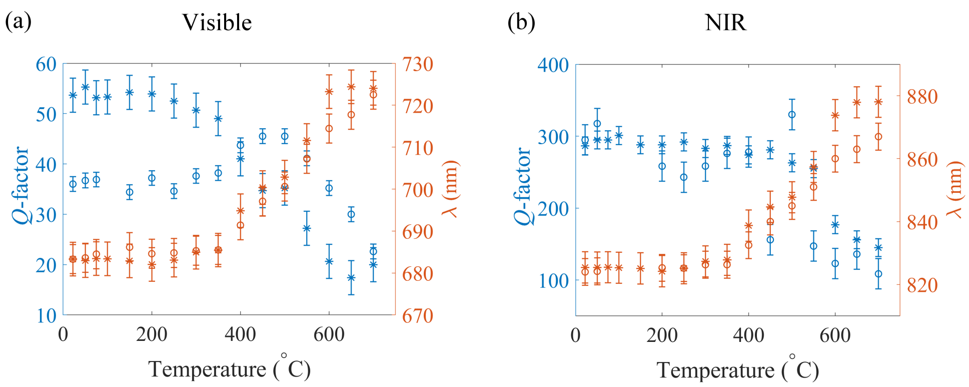

Figure S5. Resonance wavelength and *Q*-factor versus temperature in the case of the (**a**) visible metasurface and the (**b**) NIR metasurface. The '∘' represents the experimental result and the '*' represents the simulated result. The error limits are indicated as the vertical lines on the data points.

The total *Q*-factor (Q_{tot}) plotted in Figure S5 can be split into radiative *Q*-factor (Q_R) and non-radiative *Q*-factor (Q_{NR}). The radiative *Q*-factor is obtained considering no losses in the simulations. The values of the total *Q*-factor and the radiative *Q*-factor being known, the non-radiative *Q*-factor can be achieved using the equation, ${Q_{tot}}^{-1} = {Q_R}^{-1} + {Q_{NR}}^{-1}$. This equation is used to evaluate the non-radiative *Q*-factor based on the other two quantities obtained from the simulations. The radiative and non-radiative *Q*-factors computed for different temperatures are listed in the tables below. Table S1 indicates the values for the visible metasurface, and Table S2 refers to the case of the NIR metasurface.

Table S1. Radiative and non-radiative contributions to the *Q*-factor of the visible metasurface at different temperatures.

T (°C)	Q_{tot}	Q_R	Q_{NR}
22	36	82.4	63.9
300	37.6	79	71.7
450	45.5	78	109.2
600	35.2	76.7	65

Table S2. Radiative and non-radiative contributions to the *Q*-factor of the NIR metasurface at different temperatures.

T (°C)	Q_{tot}	Q_R	Q_{NR}
22	295	459	825.6
300	258	334	1134.8
450	156	210.7	600.9
600	123	131	2014.1

The equality of radiative and non-radiative *Q*-factors is important for many applications such as light absorption [2], nonlinear frequency conversion [3] etc. A reduction in the radiative *Q*-factor with the rise in the temperature is observed for both the visible and the NIR quasi-BIC structures. However the non-radiative *Q*-factor has a clear trend to increase when the temperature increases. This behavior is expected because the losses become higher at larger temperatures as observed from the ellipsometric measurements. For T = 600 °C (visible quasi-BIC structure) and T = 450 °C (NIR quasi-BIC structure), the non-radiative *Q*-factor does not increase with the temperature, this is due to the uncertainty and possible errors in the k values arising from the inaccuracy in the ellipsometric measurements, as we explained in the manuscript. The results indicate the possibility of

utilizing temperature to adjust the radiative Q-factor close to the non-radiative Q-factor for accessing the critical coupling regime.

S6. Near-field intensity pattern

The map of the Electric field was simulated for the case of T = 22 °C and T = 700 °C. Figure S6a,b represents the near-field distribution, (with the color bar representing the Electric field enhancement factors) at room temperature (T = 22 °C) for the visible and the NIR metasurfaces. The near-field map of both the metasurfaces at T = 700 °C is shown in Figure S6c,d. Except for a decrease in the enhancement factors, which corresponds to the decline in the Q-factor. The near-field distribution seems unchanged for the lowest and highest analyzed temperatures.

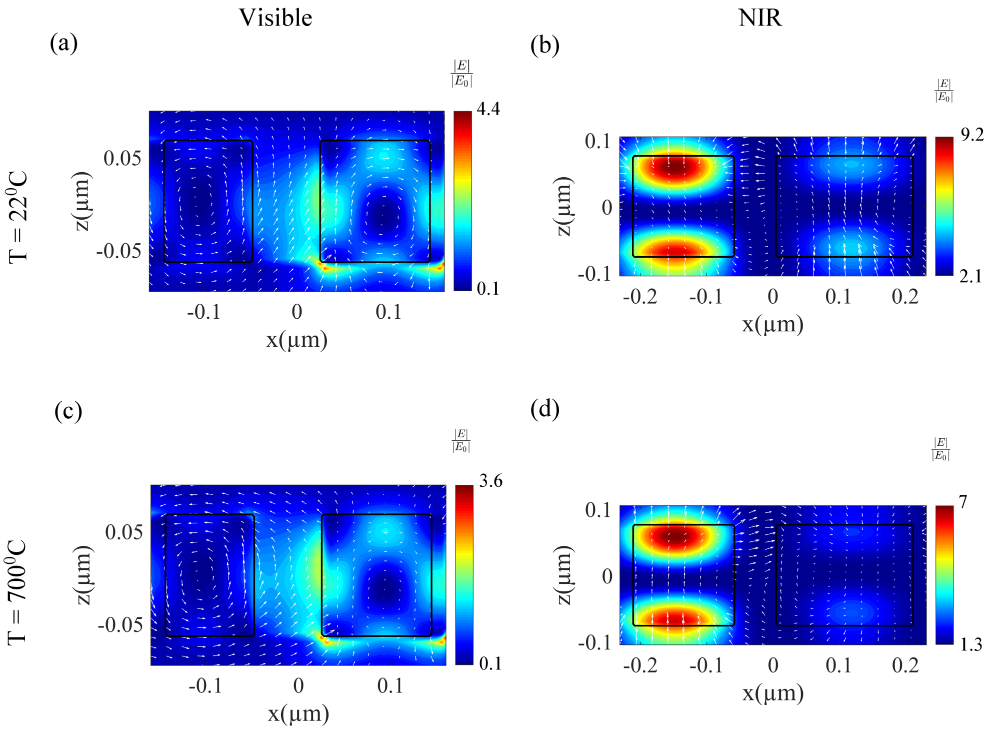

Figure S6. The near-field Electric field map (in the XZ plane through the center of the bars) at T = 22 °C of the (**a**) visible metasurface and the (**b**) NIR metasurface. Near-field Electric field map at T = 700 °C of the (**c**) visible metasurface and the (**d**) NIR metasurface.

References

1. Lumerical Solutions, Inc. Available online: http://www.lumerical.com (accessed on 15 March 2023).
2. Xiao, S.; Wang, X.; Duan, J.; Liu, T.; Yu, T. Engineering light absorption at critical coupling via bound states in the continuum. *J. Opt. Soc. Am. B* **2021**, *38*, 1325–1330.
3. Koshelev, K.; Tang, Y.; Li, K.; Choi, D.-Y.; Li, G.; Kivshar, Y. Nonlinear Metasurfaces Governed by Bound States in the Continuum. *ACS Photonics* **2019**, *6*, 1639–1644.
